# Early‐life high‐fat diet exposure increases Achilles tendon stiffness and induces transcriptomic alterations

**DOI:** 10.1002/2211-5463.70253

**Published:** 2026-04-19

**Authors:** Heyong Yin, Ziyang Yuan, Wenli Jiang, Ai Guo

**Affiliations:** ^1^ Department of Orthopaedics, Beijing Friendship Hospital Capital Medical University China; ^2^ Department of Ultrasound Chinese PLA General Hospital Beijing China

**Keywords:** Achilles tendon, extracellular matrix, high‐fat diet, tendon biomechanics, transcriptomics

## Abstract

High‐fat diet (HFD) exposure is a recognized risk factor for tendinopathy and impaired tendon healing in adults, yet its effects on baseline tendon properties following early‐life exposure remain poorly understood. In this study, we investigated the structural, biomechanical, and transcriptomic characteristics of Achilles tendons in 12‐week‐old rat offspring born to dams fed an HFD or a normal diet during gestation. Compared with controls, HFD‐exposed tendons exhibited a significant reduction in anteroposterior diameter on sagittal ultrasound imaging. However, histological assessment revealed comparable cellular density and collagen fiber organization between groups. Biomechanically, HFD exposure was associated with a significant increase in tendon stiffness, while the maximum tensile load was not significantly altered. Transcriptomic profiling identified 980 differentially expressed genes, including a marked downregulation of key tenogenic markers such as Scx and Eya2, along with an enrichment of pathways related to extracellular matrix remodeling and inflammation. Gene set enrichment further revealed an upregulation of inflammatory response, adipogenesis, and osteoblast differentiation signatures. Together, these findings demonstrate that early‐life HFD exposure induces biomechanical and molecular alterations in intact Achilles tendons, suggesting compromised tendon quality that may increase susceptibility to mechanical overload and injury later in life.

## Graphical abstract

Early‐life exposure to a high‐fat diet altered intact Achilles tendons in rat offspring, making them thinner, stiffer, and molecularly distinct even without injury. These findings suggest that developmental high‐fat diet exposure may impair tendon quality and increase susceptibility to mechanical overload or tendon injury later in life.

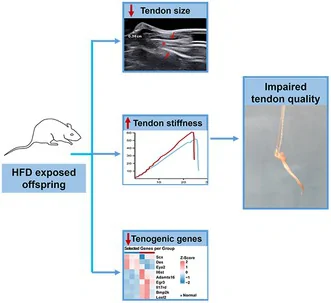

**Abbreviations**
AP: anteroposterior diameter
BP: biological process
DEGs: differentially expressed genes
Eya2: eyes absent homolog 2
GO: gene ontology
GSEA: gene set enrichment analysis
HFD: high‐fat diet
MF: molecular function
ML: mediolateral diameter
PCA: principal component analysis
Scx: Scleraxis

Tendons, mainly composed of collagen fibers and tenocytes, are designed to transmit forces between muscle and bone. And tendinopathy describes a multifaceted pathology of impaired tendon healing characterized by pain, swelling, and lower performance [1]. As for the healing process, tendons pass throughout three main phases to be repaired, named inflammation, proliferation, and remodeling. However, due to the decreased integration of collagen fibers with a higher ratio of collagen type III to collagen type I, the healed tendon cannot regain the equal mechanical properties in most cases [2]. Besides, HFD has been reported to obviously impair the tendon healing process with decreased mechanical properties [3, 4], resulting in tendons that are more prone to rupture after repair. In order to develop more effective treatment strategies, it is necessary to deeply investigate the relationship between HFD and tendon health, as well as the potential mechanisms by which metabolic diseases induce and exacerbate tendinopathy.

Previous studies have indicated that metabolic diseases adversely affect tendon repair [5, 6, 7]. Recent research has also investigated the impact of an HFD on adult tendon tissue, revealing that animals subjected to HFD exhibited metabolic disorders that resulted in detrimental effects on the mechanical properties of tendons [8, 9]. In parallel, most of the available literature has focused on injured or healing tendons, in which HFD or diabetes impairs matrix remodeling, reduces mechanical competence, and compromises repair quality [3, 4]. By comparison, studies examining intact tendons, particularly after maternal or early‐life dietary exposure, remain limited. Bolam et al. demonstrated that maternal HFD during pregnancy significantly increased rotator cuff elasticity in male offspring [10]. Furthermore, David et al. reported that tendons from HFD‐fed rats displayed impaired biomechanical performance at 28‐day postinjury [3]. Interestingly, Studentsova et al. found that rats fed an HFD for 12 weeks, followed by a transition to a low‐fat diet, experienced greater impairments in tendon gliding function compared to those continuously maintained on an HFD [8]. However, its effects on intact Achilles tendon structure, biomechanics, and molecular characteristics following early‐life HFD exposure have not yet been elucidated. Accordingly, the novelty of the present work lies in evaluating the baseline phenotype of an uninjured weight‐bearing tendon after developmental HFD exposure, rather than the response of an already injured tendon to a metabolically adverse milieu.

The present study aimed to investigate the impact of early‐life HFD exposure on the biomechanical, structural, and transcriptomic characteristics of intact Achilles tendons in offspring. Ultrasonography, histological analysis, and biomechanical testing were employed to evaluate tendon morphology and mechanical behavior, while bulk RNA sequencing was used to comprehensively characterize transcriptional alterations and identify differentially expressed genes between HFD‐exposed and control groups. We hypothesized that early‐life HFD exposure would lead to changes in tendon mechanical properties and gene expression profiles, reflecting compromised tendon quality and a potential increase in susceptibility to mechanical overload and injury later in life.

## Materials and methods

### Animal model and study design

Female Sprague–Dawley rats were housed under controlled environmental conditions with a 12‐h light/dark cycle and free access to food and water. Dams were fed either a high‐fat diet (60% kcal fat, 20% carbohydrate, 20% protein) or standard chow throughout gestation and lactation. Male offspring therefore experienced the maternal dietary intervention during both fetal development and the preweaning suckling period. Offspring were weaned at postnatal day 21, which is the standard time of nutritional independence in rats, and were then maintained on a standard diet until 12 weeks of age to isolate the lasting effect of early‐life exposure from continued direct HFD intake after weaning. At 12 weeks, male offspring were randomly selected for Achilles tendon analysis. To eliminate the potential influence of sex differences, only male offspring were included in the analyses. Following anesthesia and euthanasia with intraperitoneal injection of 3% pentobarbital sodium, the skin and fascia of the hindlimb were removed, and the Achilles tendon complex was carefully dissected free of surrounding soft tissues; visible muscle remnants were trimmed as thoroughly and consistently as possible before downstream analyses. All animal procedures were approved by the Ethics Committee of Beijing Friendship Hospital, Capital Medical University (Approval No. 21‐1018; approved on 18 August 2021) and are reported in accordance with the ARRIVE 2.0 guidelines, including the Essential 10.

### Ultrasound examination

Ultrasound examination of the Achilles tendon (*n* = 4) was performed using a high‐frequency linear transducer (SL15‐4, Aixplorer, Supersonic Imagine, France). All measurements were obtained under standardized conditions with the animals positioned prone and the ankle in neutral position to minimize tendon tension. Both longitudinal (sagittal) and transverse (cross‐sectional) planes were scanned. On longitudinal (sagittal) images, the anteroposterior diameter (AP) of the Achilles tendon was measured as the distance between the anterior and posterior borders of the tendon at its thickest region. On transverse (cross‐sectional) images, the mediolateral diameter (ML) was determined as the distance between the medial and lateral borders. All scans were performed by the same experienced operator (W. Jiang) to minimize interobserver variability.

### Histology

Achilles tendons designated for histological analysis (*n* = 3) were fixed in 4% paraformaldehyde for 24 h at room temperature. After fixation, tissues were dehydrated through a graded ethanol series (70%, 80%, 90%, and 100%, 2 h each), cleared in n‐butanol for 12 h, and embedded in paraffin for 6 h. Serial longitudinal sections of 3 μm thickness were obtained and stained with hematoxylin and eosin (H&E) for morphological evaluation. Stained sections were digitized using a slide scanner (Pannoramic MIDI, 3DHISTECH, Hungary) and analyzed with CaseViewer 2.0 software. Cell density (cells·mm^−2^) was quantified within defined regions of interest. To assess cellular alignment, the tenocyte nuclear angle deviation was measured as the angle between the longitudinal axis of the Achilles tendon and the major axis of the tenocyte nucleus, using the ‘Angular’ tool of AxioVision software v4.8 (Carl Zeiss, Jena, Germany), as previously described [11]. Because tenocytes are typically oriented along the collagen fibers, the degree of tenocyte alignment also served as an indirect indicator of collagen fiber organization.

### Biomechanical testing

Biomechanical testing (*n* = 8) was performed to assess the mechanical properties of the Achilles tendons. The Achilles tendon complex, including the gastrocnemius muscle and calcaneal insertion, was carefully dissected. Samples were stored in 0.9% saline at −80 °C until testing. Prior to testing, tendons were thawed to room temperature and kept moist throughout the procedure to prevent dehydration. Each specimen was mounted on a mechanical testing system and subjected to a passive preload ranging from 0.0 to 0.5 N to eliminate slack. Subsequently, the tendons were stretched at a constant displacement rate of 150 mm·min^−1^ until failure. The maximum load was defined as the peak value of the force–displacement curve, and stiffness was calculated as the slope of the linear region of the curve, representing the elastic response of the tissue.

### RNA library construction, RNA sequencing, and qPCR validation

Total RNA was extracted from the tendon tissues (*n* = 3) in each group. RNA concentration and purity were measured using NanoDrop 2000 Spectrophotometer (Thermo Fisher Scientific, Wilmington, DE, USA). RNA integrity was assessed using the RNA Nano 6000 Assay Kit of the Agilent Bioanalyzer 2100 system (Agilent Technologies, Santa Clara, CA, USA). The construction of sequencing libraries was performed by Biomarker Technologies Corporation (Biomarker Technologies Corporation, Beijing, China), which were generated using NEBNext UltraTM RNA Library Prep Kit for Illumina (NEB, Ipswich, MA, USA) based on the manufacturer's recommendations. Index codes were added to attribute sequences to each sample. The clustering of the index‐coded samples was carried out on a cBot Cluster Generation System using TruSeq PE Cluster Kit v4‐cBot‐HS (Illumina, San Diego, CA, USA), following the manufacturer's instructions. After cluster generation, the library preparations were sequenced on an Illumina NovaSeq 6000 and paired‐end reads were generated. Clean reads were obtained by removing adapter‐containing reads, poly‐N reads, and low‐quality reads. Clean reads were mapped to the reference genome using HISAT2. Gene expression levels were estimated as fragments per kilobase of transcript per million mapped reads (FPKM) for visualization. Differential expression analysis was performed on raw read counts using the DESeq2 package in R [12]. Significant DEGs were identified using the following thresholds: false discovery rate (FDR) < 0.05 and fold change (FC) > 2 or < 0.5.

Quantitative real‐time PCR (qPCR) (*n* = 3) was conducted to validate the expression of four DEGs, namely Scx, Eya2, Il6st, and Loxl2, using the iCycler iQ™ Real‐Time PCR Detection System (Bio‐Rad, Hercules, CA, USA). Relative mRNA expression levels were normalized to glyceraldehyde‐3‐phosphate dehydrogenase (GAPDH) and determined using the $2^{-\Delta \Delta C_{t}}$ method. The primer sequences employed for qPCR analysis are listed in Table S1.

### Statistical analysis

All statistical analyses for ultrasound measurements, histological quantification, biomechanical parameters, and qPCR validation were performed using graphpad prism 8.0 (GraphPad Software, La Jolla, CA, USA). These data are presented as mean ± standard deviation (SD), unless otherwise indicated in the corresponding figure legends. Comparisons between two groups were conducted using an unpaired two‐tailed Student's *t*‐test. Differential expression analysis of RNA‐seq data was performed on raw read counts using the DESeq2 package in R. Genes with a false discovery rate (FDR) < 0.05 and fold change > 2 or < 0.5 were considered differentially expressed. A *P* value of < 0.05 was considered statistically significant.

## Results

### HFD exposure alters Achilles tendon morphology on ultrasound imaging

**Fig. 1:**
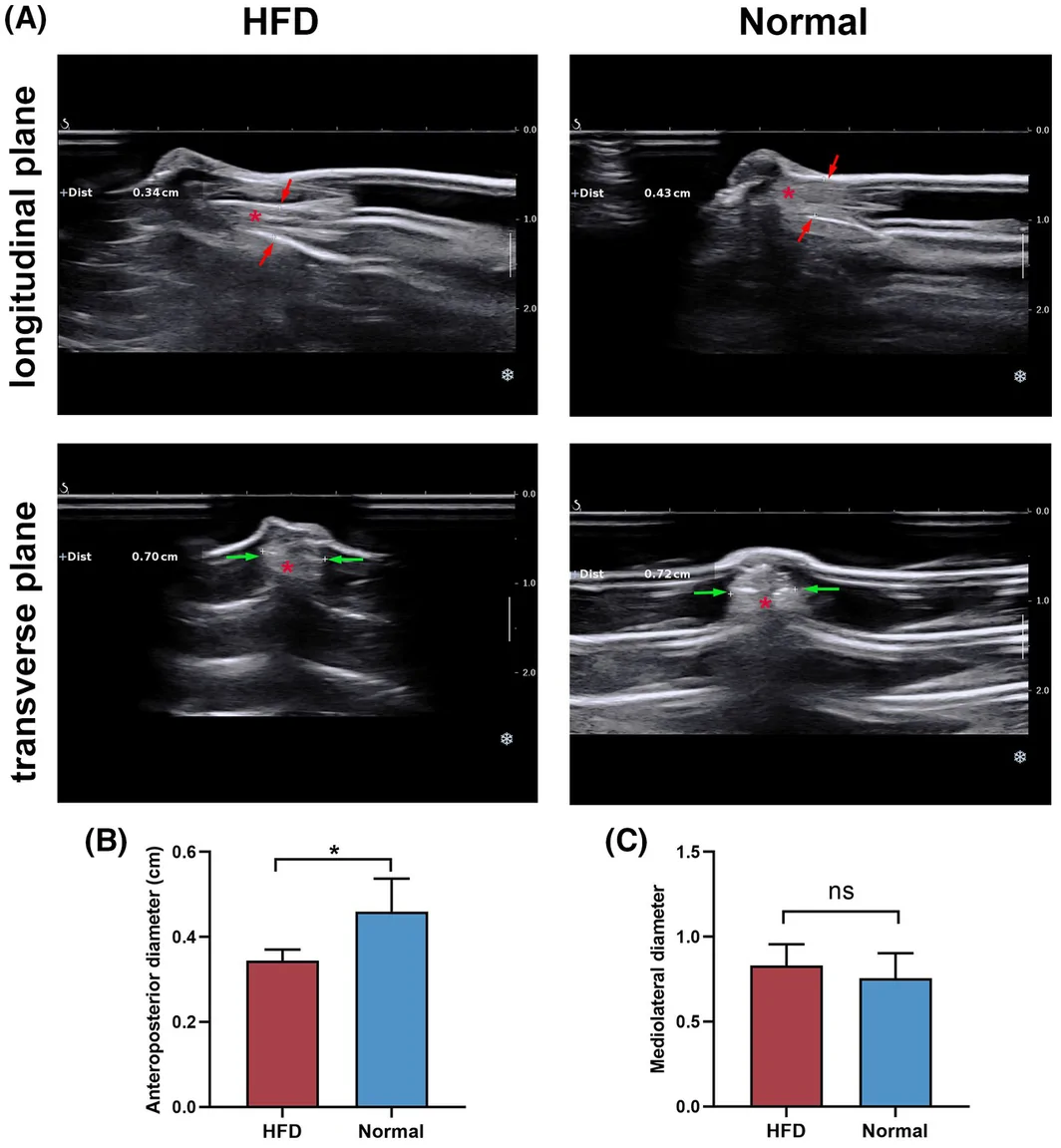
Ultrasound morphological assessment of the Achilles tendon (*n* = 4 per group). (A) Representative longitudinal and transverse ultrasound images of the Achilles tendon in the HFD and Normal groups. Red stars indicate the Achilles tendon; red arrows denote the anterior and posterior borders, while green arrows indicate the medial and lateral borders. The depth scale shown on the right side of each ultrasound image is in centimeters, and the vertical white bar indicates 0.5 cm. (B) The anteroposterior diameter (AP) of the Achilles tendon was measured as the distance between the anterior and posterior borders at the thickest region. (C) The mediolateral diameter (ML) was determined as the distance between the medial and lateral borders on transverse images. Data are presented as mean ± SD. Statistical comparisons were performed using an unpaired two‐tailed Student's *t*‐test. *P < 0.05, **P < 0.01, ***P < 0.001, and ns indicates no significant difference.

High‐resolution ultrasonography was employed to assess Achilles tendon morphology in offspring from both groups (Fig. 1A). Quantitative analysis revealed that tendons from the HFD group displayed a markedly smaller anteroposterior (AP) diameter on sagittal views compared with those from the Normal group (HFD: 0.35 ± 0.025 cm vs. Normal: 0.46 ± 0.077 cm, *P* < 0.05) (Fig. 1B). In contrast, no significant difference was observed in the mediolateral (ML) diameter on transverse images (HFD: 0.83 ± 0.13 cm vs. Normal: 0.76 ± 0.15 cm, *P* > 0.05) (Fig. 1C). The selective reduction in AP diameter, with preserved ML dimension, suggests a morphological alteration characterized by tendon thinning or flattening rather than an overall size decrease.

### HFD exposure does not significantly affect histological tendon architecture

**Fig. 2:**
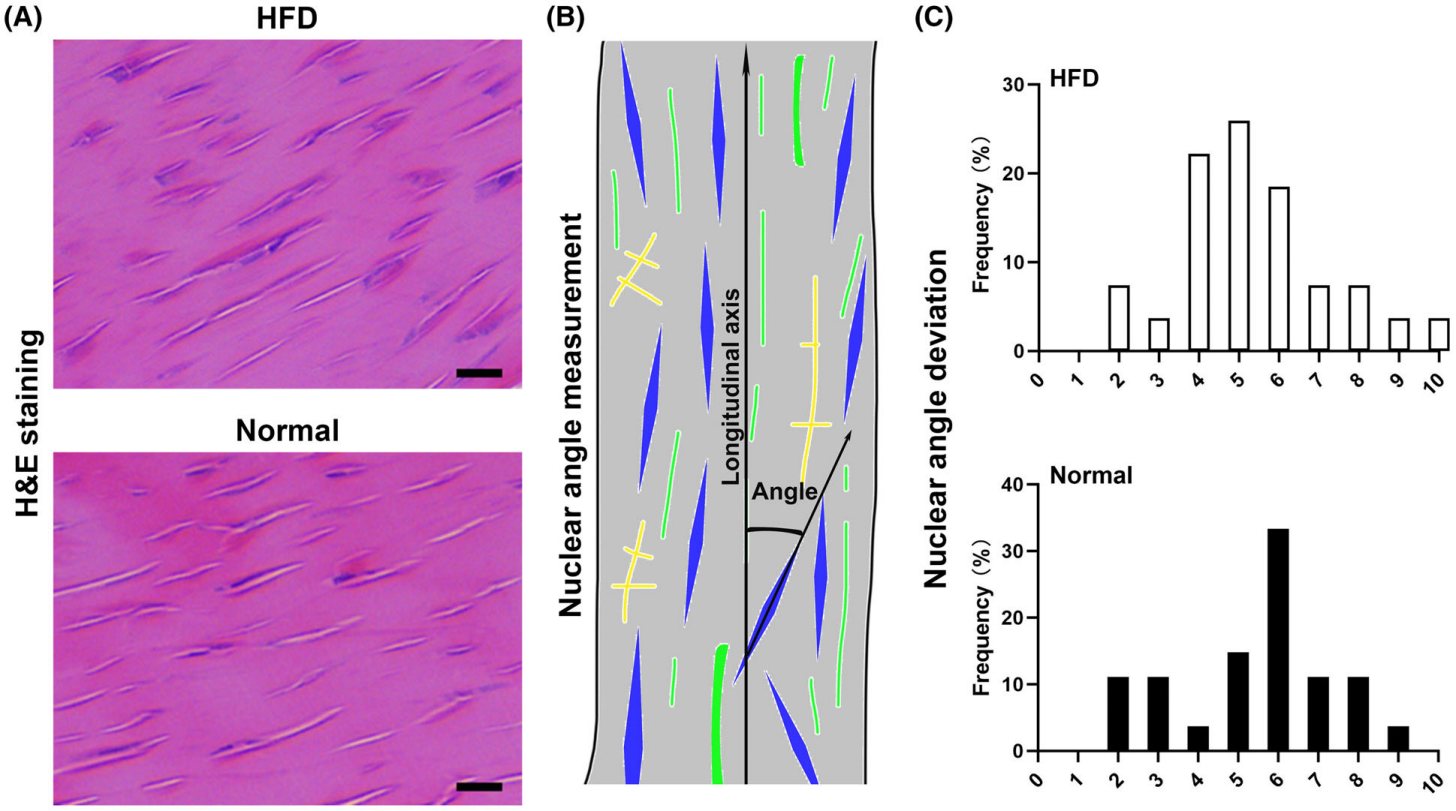
Histological evaluation of Achilles tendon structure (*n* = 3 per group). (A) Representative hematoxylin and eosin (H&E) staining images of the Achilles tendon in the HFD and Normal groups. Scale bar: 20 μm. (B) Schematic illustration of tenocyte nuclear angle deviation, defined as the angle between the longitudinal axis of the Achilles tendon and the major axis of the tenocyte nucleus. (C) Frequency distribution of nuclear angle deviation in the HFD and Normal groups.

H&E staining revealed a comparable overall microarchitecture of the Achilles tendon between offspring from HFD and normal dams (Fig. 2A). Quantitative analysis showed no significant difference in tenocyte density between groups (HFD: 1079 ± 114 cells·mm^−2^ vs. Normal: 1026 ± 111 cells·mm^−2^; *P* > 0.05). In addition, tenocyte nuclear angle deviation, defined as the angle between the long axis of the nucleus and the longitudinal axis of the Achilles tendon, was assessed to evaluate fiber alignment (Fig. 2B). The results demonstrated a similar degree of nuclear alignment in both groups, with 92.6% of nuclei in the HFD group and 96.3% in the Normal group deviating by 2°–8° from the tendon axis (Fig. 2C), indicating that the overall collagen fiber alignment was not notably affected by early‐life HFD exposure.

### HFD exposure increases the mechanical stiffness of Achilles tendons

**Fig. 3:**
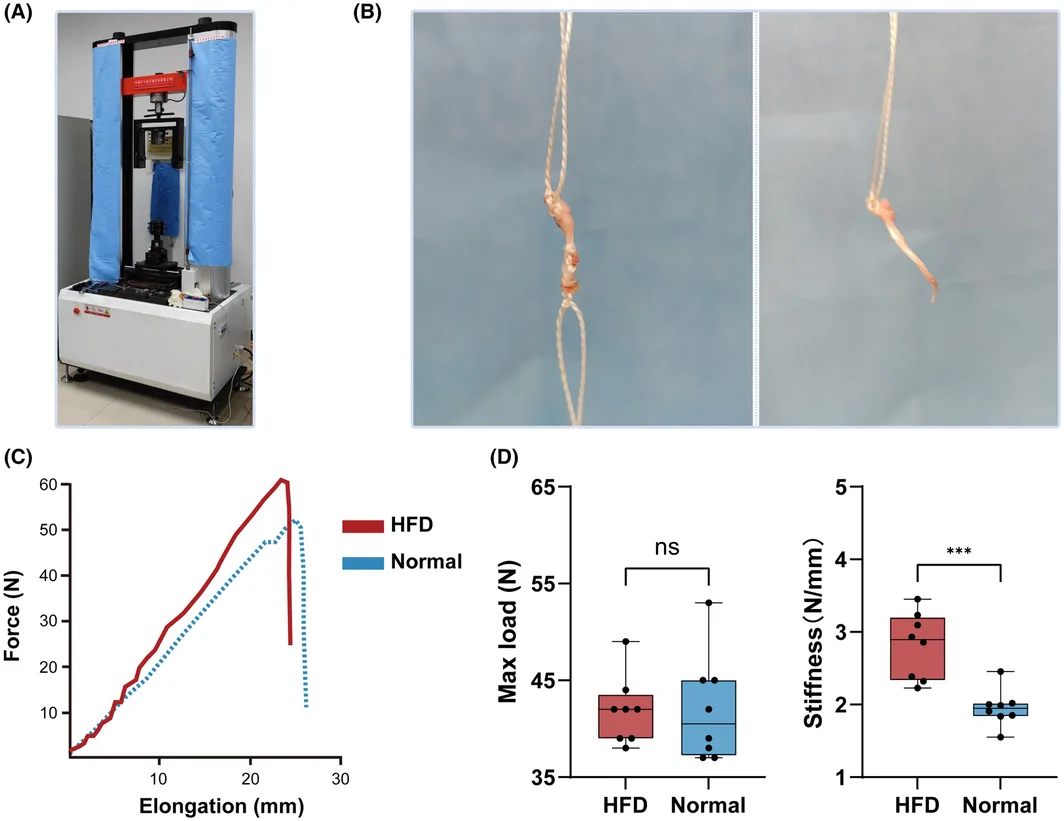
Biomechanical assessment of Achilles tendons (*n* = 8 per group). (A) The biomechanical testing platform. (B) Tendons were mounted and stretched at a constant displacement rate of 150 mm·min^−1^ until failure. (C) Representative force–elongation curves from each group. (D) Comparison of maximum load and stiffness between groups. The box represents the interquartile range, the center line indicates the median, and the whiskers extend to the minimum and maximum values. Individual dots represent individual samples. Statistical comparisons were performed using an unpaired two‐tailed Student's *t*‐test. *P < 0.05, **P < 0.01, ***P < 0.001, and ns indicates no significant difference.

Biomechanical testing was conducted to assess the mechanical properties of the Achilles tendons from offspring in both groups (Fig. 3A). The tendons were mounted and stretched at a constant displacement rate of 150 mm·min^−1^ until failure (Fig. 3B,C). The maximum tensile load did not differ significantly between the two groups. Specifically, the ultimate load was 41.86 ± 3.52 N in the HFD group and 42.00 ± 5.53 N in the Normal group (*n* = 8, *P* > 0.05) (Fig. 3D). In contrast, tendon stiffness was markedly increased in the HFD group, measuring 2.81 ± 0.45 N·mm^−1^ compared with 1.95 ± 0.25 N·mm^−1^ in the Normal group (*P* < 0.001) (Fig. 3D). Taken together, these findings indicate that while early‐life HFD exposure did not alter the ultimate tensile strength of the offspring Achilles tendon, it led to a significant increase in stiffness.

### HFD exposure induces transcriptomic alterations in Achilles tendons

**Fig. 4:**
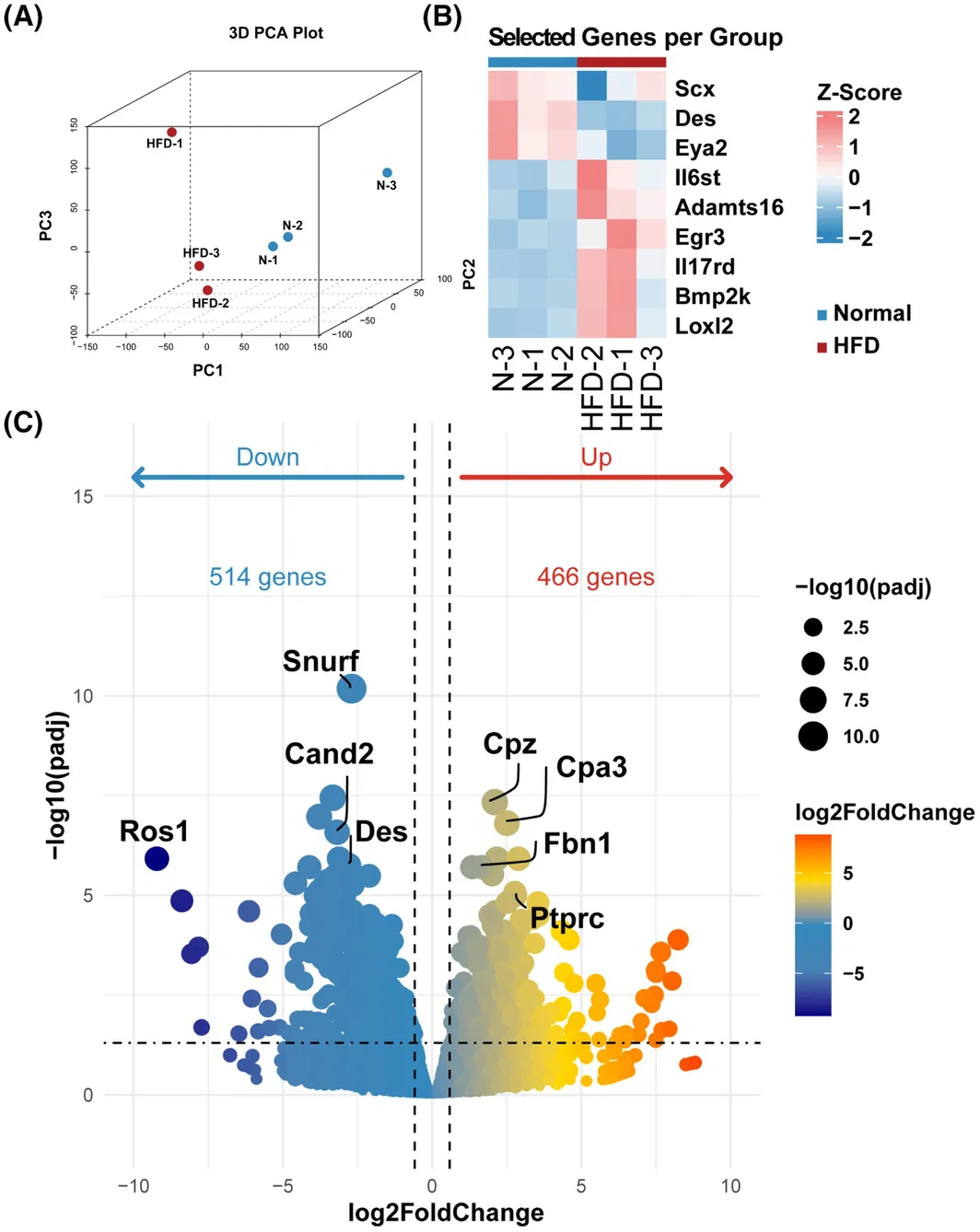
Transcriptomic analysis of Achilles tendons from the HFD and Normal dams (*n* = 3 per group). (A) Three‐dimensional principal component analysis (PCA) showing the clustering of samples from each group. (B) Heatmap of selected differentially expressed genes (DEGs) exhibiting increasing or decreasing trends. Each column represents an individual sample. (C) Volcano plot depicting DEGs between the HFD and Normal groups; each dot corresponds to a single gene.

A total of 6 samples were processed for transcriptome sequencing, and principal component analysis (PCA) was conducted to visualize the sample relationships. The variation caused by the first two principal components (PC1 and PC2) successfully separated two clusters based on the gene expression profile of the samples from the two groups (Fig. 4A). Based on the expression levels of DEGs, we performed hierarchical clustering analysis between the HFD and normal groups. Among the DEGs, several genes were closely associated with tendon development, including Scx, a transcription factor required for tenogenesis [13]. We therefore highlighted representative tendon‐related genes in a heatmap (Fig. 4B). Des was also more highly expressed in the normal group. Because Des is a muscle‐enriched intermediate filament gene that maintains the structural integrity of skeletal muscle [14], this signal may partly reflect minor residual tissue from the muscle–tendon junction or surrounding soft tissue in bulk Achilles tendon dissections. Importantly, the overall transcriptomic differences between groups were not limited to Des and included multiple tendon‐ and matrix‐relevant genes, such as Scx, Eya2, Il6st, Il17rd, Adamts16, and Loxl2, together with enrichment of extracellular matrix organization, collagen fibril organization, and glycosaminoglycan‐binding pathways. Meanwhile, several inflammatory markers, such as Il6st, Egr3, and Il17rd, were significantly upregulated in the HFD group. We further validated the expression of four representative genes (Scx, Eya2, Il6st, and Loxl2) by qPCR, and the results were consistent with the RNA‐seq data (Fig. S1). Subsequently, we undertook a deeper exploration of the two groups in terms of DEGs, and a total of 980 genes were differentially expressed, with 466 and 514 DEGs upregulated and downregulated, respectively, which revealed significant differences in gene expression between two groups (Fig. 4C).

### HFD is associated with enrichment of inflammatory and extracellular matrix related pathways

**Fig. 5:**
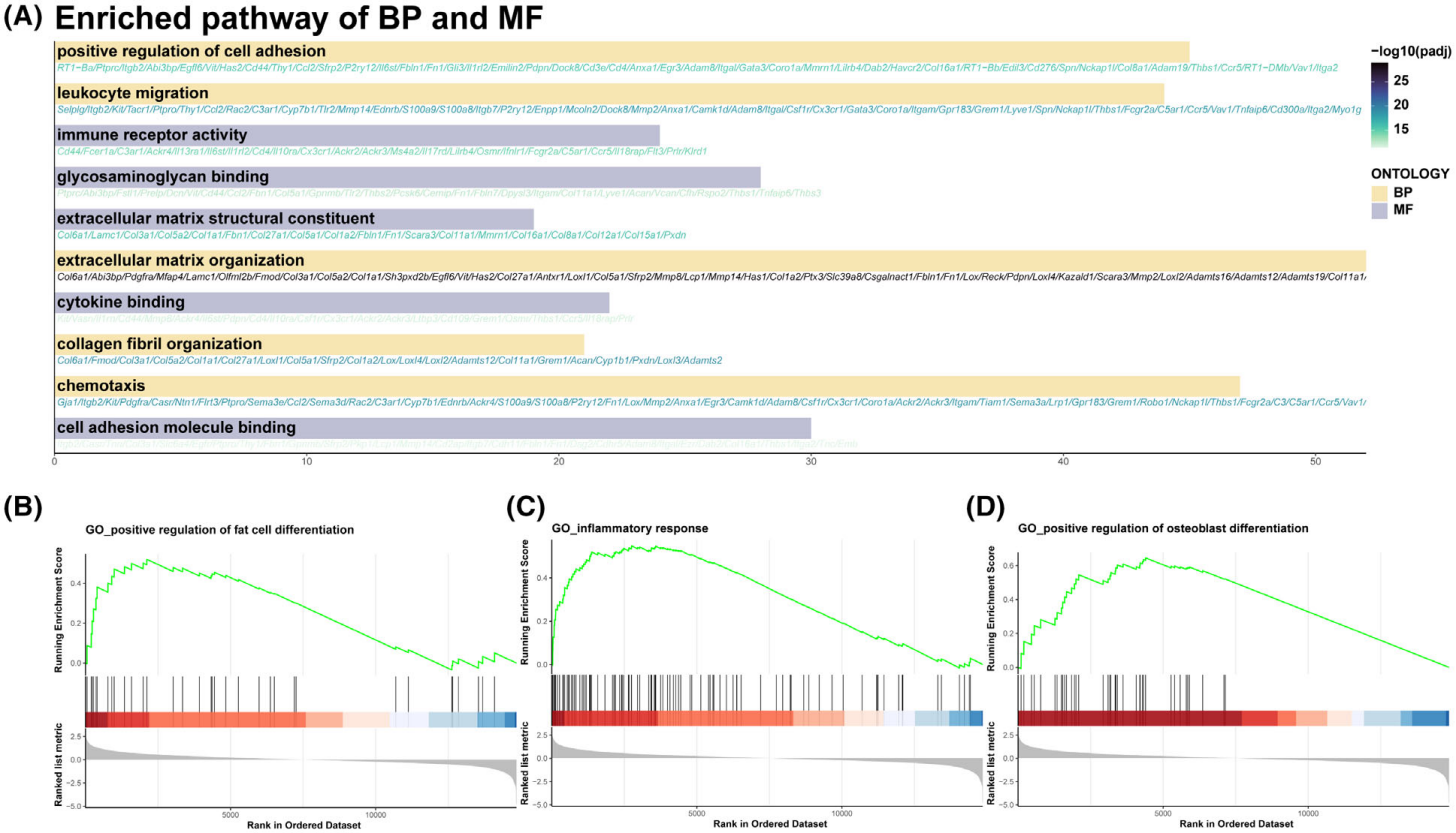
Functional annotation and pathway analysis of DEGs in offspring Achilles tendons (*n* = 3 per group). (A) GO analysis showing enriched biological processes (BP) and molecular functions (MF) of DEGs. (B–D) Gene Set Enrichment Analysis (GSEA) highlighting representative pathways enriched in the HFD group, including positive regulation of fat cell differentiation, inflammatory response, and positive regulation of osteoblast differentiation.

Functional annotation of DEGs was performed using gene ontology (GO) analyses with terms involved in biological process (BP) and molecular function (MF). As shown in Fig. 5A, DEGs involved in the BP were mainly related to positive regulation of cell adhesion, leukocyte migration, extracellular matrix organization, collagen fibril organization, and chemotaxis. DEGs enriched in MF terms were mainly related to immune receptor activity, glycosaminoglycan binding, extracellular matrix structural constituent, cytokine binding, and cell adhesion molecule binding. Furthermore, we analyzed the representative pathways of highly expressed DEGs using gene set enrichment analysis (GSEA) and found that the ones with HFD had a relation to tendon healing, including positive regulation of fat cell differentiation, inflammatory response, and positive regulation of osteoblast differentiation (Fig. 5B–D).

## Discussion

HFD has increasingly been recognized as a significant risk factor for tendinopathy and impaired tendon healing [15]. Most previous experimental work in this area has focused on injured or healing tendons, showing that metabolic disturbance compromises repair quality, collagen remodeling, and postinjury mechanical performance [3, 4]. By contrast, the present study was designed to examine intact Achilles tendons, thereby addressing whether early‐life metabolic exposure is sufficient to alter the baseline tendon phenotype before any superimposed injury. To our knowledge, this is the first study to demonstrate that early‐life HFD exposure alters the structural, biomechanical, and transcriptomic characteristics of intact Achilles tendons *in vivo*. Previous clinical studies have reported that obesity is associated with reduced joint mobility, a higher risk of tendon rupture, and poorer outcomes following tendon repair surgery [16, 17, 18]. Consistent with these observations, our findings indicate that early‐life dietary exposure can influence baseline tendon structure and mechanical behavior. Specifically, the HFD group exhibited a reduced anteroposterior diameter of the Achilles tendon and a significant increase in tendon stiffness at 12 weeks of age, despite a comparable maximum tensile load relative to controls. At the molecular level, transcriptomic profiling revealed downregulation of key tenogenic markers and enrichment of inflammation‐related pathways. Notably, histological architecture remained largely preserved, suggesting that the observed early tendon phenotype reflects subtle biomechanical and transcriptional adaptations rather than overt structural degeneration.

In the biomechanical tests, tendons from the HFD offspring displayed increased stiffness without alteration in maximum tensile load, indicating that the tendons became less compliant and more resistant to deformation under load. This mechanical behavior suggests that HFD exposure during gestation may have induced a stiffer, less extensible tendon matrix. The increase in stiffness could be attributed to alterations in extracellular matrix (ECM) remodeling, such as excessive collagen cross‐linking, changes in collagen fibril organization, or shifts in the composition of noncollagenous matrix components. Such changes may lead to reduced tendon elasticity and, consequently, increased susceptibility to rupture or microdamage under repetitive mechanical stress in adulthood. This observation is in line with previous reports indicating that tendons from obese or metabolically challenged animals exhibit higher stiffness and altered mechanical integrity over time [3, 8].

Several studies have explored the relationship between metabolic disturbances and tendon biomechanics. David et al. found no significant differences in stiffness or maximum force between HFD and control rats at 12 weeks [3], while Studentsova et al. demonstrated that stiffness was increased at 24 weeks and the ultimate tensile load was significantly decreased at 48 weeks in the HFD group compared to the low‐fat diet group [8]. These findings suggest that the mechanical consequences of HFD on tendon tissues are progressive and time‐dependent. Our data, showing increased stiffness but unchanged maximum load at 12 weeks, likely reflect an early‐stage phenotype, where biochemical and microstructural remodeling precedes overt mechanical failure. It is reasonable to infer that longer exposure or aging may exacerbate these alterations, ultimately compromising tendon strength. Our model also differs conceptually from studies using direct postweaning high‐fat diet feeding, because it captures early‐life metabolic programming by maternal diet during gestation and lactation. The study was designed to evaluate the combined effects of maternal high‐fat diet exposure across these periods, rather than to distinguish prenatal from postnatal influences. As such, it reflects a biologically relevant developmental programming paradigm. However, the relative contributions of prenatal and lactational exposure cannot be resolved in the present design and should be addressed in future studies using time‐restricted maternal dietary interventions.

Regarding tendon morphology, Tomlinson et al. reported that individuals with higher BMI had a larger Achilles tendon cross‐sectional area, although no significant association was observed between BMI and Young's modulus [19]. Similarly, other studies found that obesity was correlated with an enlarged tendon CSA in adults [20]. In our study, early‐life HFD exposure resulted in a thinner (reduced anteroposterior diameter) but not wider (mediolateral diameter unchanged) tendon, suggesting a shape remodeling rather than uniform hypertrophy. This morphological change might represent an early adaptive response to altered matrix tension or loading environment during development.

At the molecular level, transcriptomic profiling identified 980 differentially expressed genes (DEGs) between HFD and normal diet offspring. Several tenogenic genes essential for tendon differentiation and maintenance were significantly downregulated, including Scx and Eya2. Scx is a key transcription factor regulating tenocyte differentiation and tendon maturation, and its suppression may impair proper ECM synthesis and fibril organization. Eya2, largely restricted to limb extensor tendons, is involved in tendon morphogenesis [21]; its downregulation may further contribute to defective tendon patterning. In parallel, several inflammation‐ and remodeling‐related genes were upregulated in the HFD group, such as Il6st [22], Egr3 [23], and Loxl2 [24], indicating active inflammatory signaling and enhanced collagen cross‐linking. The simultaneous upregulation of Il17rd, which negatively regulates inflammation [25], suggests the presence of a complex, compensatory inflammatory network. Moreover, increased expression of Adamts16, involved in ECM degradation [26], and activation of BMP signaling, known to promote tenocyte calcification [27], imply a tendency toward matrix disorganization and even heterotopic ossification.

Functional annotation through GO and GSEA provided further insight into the biological alterations. GO analysis revealed enrichment of genes associated with ECM remodeling (e.g., glycosaminoglycan binding, ECM organization) and inflammatory response, suggesting that early‐life HFD exposure may reprogram the tendon microenvironment toward a more fibrotic and inflammatory state. GSEA showed significant enrichment in pathways related to fat cell differentiation, inflammatory signaling, and osteoblast differentiation. Notably, adipocyte accumulation is a characteristic feature of degenerative tendons and is often resistant to both surgical and rehabilitative treatments [28]. Chronic low‐grade inflammation has been reported to impair tendon healing [29], while excessive osteogenic signaling can lead to heterotopic ossification, contributing to tendon stiffness and recurrent injuries [30, 31]. Together, these transcriptomic signatures suggest that early‐life HFD exposure may predispose tendons to a maladaptive phenotype characterized by inflammation‐driven matrix remodeling, increased stiffness, and potential ossification risk.

Nevertheless, several limitations of this study should be acknowledged. First, although the transcriptomic analyses revealed significant alterations in pathways related to inflammation and tenogenic regulation, further validation at the protein and cellular levels is needed to clarify the underlying mechanisms. In addition, because RNA‐seq was performed on bulk Achilles tendon preparations, minor contributions from adjacent nontendinous tissues at the muscle–tendon interface cannot be completely excluded. However, the concordant changes in established tendon‐related genes, together with enrichment of extracellular matrix organization and collagen fibril organization pathways, support the biological relevance of the tendon‐associated transcriptional response. Finally, longitudinal studies and injury models will be necessary to determine whether these early biomechanical and molecular alterations increase susceptibility to tendon degeneration or impaired healing later in life.

## Conclusion

In summary, early‐life HFD exposure adversely affects the structure, biomechanics, and transcriptomic profile of intact Achilles tendons, as evidenced by increased tissue stiffness, reduced tendon thickness, and molecular alterations associated with inflammation and impaired tenogenic signaling. Although histological architecture remained largely preserved, the observed biomechanical and molecular changes suggest a latent vulnerability of intact tendons to mechanical overload and degeneration. These findings indicate the potential impact of early‐life metabolic challenges on tendon quality and highlight the importance of considering dietary influences in musculoskeletal health research.

## Conflict of interest

The authors declare no conflict of interest.

## Author contributions

HY and AG conceived and designed the study. HY, ZY, and WJ performed the experiments, analyzed the data, and drafted the manuscript. All authors reviewed, revised, and approved the final version of the manuscript.

## Supporting information


**Fig. S1.** Quantitative PCR validation of the expression levels of four representative differentially expressed genes (Scx, Eya2, Il6st, and Loxl2).
**Table S1.** Primer sequences used for quantitative real‐time PCR analysis.

## Data Availability

The RNA sequencing data generated in this study are available in the NCBI repository under accession number PRJNA1264475. All other data supporting the findings of this study are provided within the article and its Supporting Information or are available from the corresponding author (guoaij@139.com) upon reasonable request.
