# Supplementary material for: Early‐life high‐fat diet exposure increases Achilles tendon stiffness and induces transcriptomic alterations
Source: FEBS Open Bio. 2026 Apr 19;16(9):1741–51. doi: 10.1002/2211-5463.70253 (PMC13398776; doi:10.1002/2211-5463.70253)
Supplement: Supplementary file 1 [file FEB4-16-1741-s001.docx]

**Table S1.** **Quantitative real‑time PCR primer sequences**

| **Target gene** | **Primers** | **Accession No** |
| --- | --- | --- |
| **Scx** | F 5’-GCACCTTCTGCCTCAGCAAC-3’  R5’-TTCTGTCACGGTCTTTGCTCA-3’ | NM_001130508.1 |
| **Eya2** | F 5’-ATTTGGGAGCGTGCACCAGGAT-3’  R5’-GCGGGTTGTATGATGGGCTGAA-3’ | NM_130427.1 |
| **Il6st** | F 5’-TGATGTCCAGAACGGATTCA-3’  R5’-GTGTATGCTGCCATGTGGAC-3’ | NM_001008725.3 |
| **Loxl2** | F 5’-CGATTGCCACCTCCTTGCTA-3’  R5’-CCAGAGCCCTGCCCCTAA-3’ | NM_001106047.2 |
| **GAPDH** | F 5’-CCGCATCTTCTTGTGCAGTG-3’  R5’-ACCAGCTTCCCATTCTCAGC-3’ | NM_017008.4 |

Fig. S1. Quantitative PCR validation of the expression levels of four representative differentially expressed genes (Scx, Eya2, Il6st, and Loxl2) (n = 3 per group). Data are presented as means ± SD. * p < 0.05, ** p < 0.01, *** p < 0.001, and ns indicates no significant difference.
